# Genome-Wide Transcriptional Response of *Silurana* (*Xenopus*) *tropicalis* to Infection with the Deadly Chytrid Fungus

**DOI:** 10.1371/journal.pone.0006494

**Published:** 2009-08-04

**Authors:** Erica Bree Rosenblum, Thomas J. Poorten, Matthew Settles, Gordon K. Murdoch, Jacques Robert, Nicole Maddox, Michael B. Eisen

**Affiliations:** 1 Department of Biological Sciences, University of Idaho, Moscow, Idaho, United States of America; 2 Departments of Molecular and Cell Biology and Integrative Biology, University of California, Berkeley, California, United States of America; 3 School of Molecular Bioscience and Department of Animal Sciences, Washington State University, Pullman, Washington, United States of America; 4 Department of Animal and Veterinary Science, University of Idaho, Moscow, Idaho, United States of America; 5 Department of Microbiology and Immunology, University of Rochester Medical Center, Rochester, New York, United States of America; 6 Howard Hughes Medical Institute, University of California, Berkeley, California, United States of America; University College Dublin, Ireland

## Abstract

Emerging infectious diseases are of great concern for both wildlife and humans. Several highly virulent fungal pathogens have recently been discovered in natural populations, highlighting the need for a better understanding of fungal-vertebrate host-pathogen interactions. Because most fungal pathogens are not fatal in the absence of other predisposing conditions, host-pathogen dynamics for deadly fungal pathogens are of particular interest. The chytrid fungus *Batrachochytrium dendrobatidis* (hereafter *Bd*) infects hundreds of species of frogs in the wild. It is found worldwide and is a significant contributor to the current global amphibian decline. However, the mechanism by which *Bd* causes death in amphibians, and the response of the host to *Bd* infection, remain largely unknown. Here we use whole-genome microarrays to monitor the transcriptional responses to *Bd* infection in the model frog species, *Silurana* (*Xenopus*) *tropicalis*, which is susceptible to chytridiomycosis. To elucidate the immune response to *Bd* and evaluate the physiological effects of chytridiomycosis, we measured gene expression changes in several tissues (liver, skin, spleen) following exposure to *Bd*. We detected a strong transcriptional response for genes involved in physiological processes that can help explain some clinical symptoms of chytridiomycosis at the organismal level. However, we detected surprisingly little evidence of an immune response to *Bd* exposure, suggesting that this susceptible species may not be mounting efficient innate and adaptive immune responses against *Bd*. The weak immune response may be partially explained by the thermal conditions of the experiment, which were optimal for *Bd* growth. However, many immune genes exhibited *decreased* expression in *Bd*-exposed frogs compared to control frogs, suggesting a more complex effect of *Bd* on the immune system than simple temperature-mediated immune suppression. This study generates important baseline data for ongoing efforts to understand differences in response to *Bd* between susceptible and resistant frog species and the effects of chytridiomycosis in natural populations.

## Introduction

Emerging infectious diseases are receiving increasing attention for their role in precipitous population declines and extinctions in natural populations (e.g., [1]). Historically, fungal pathogens of wildlife have been understudied because they are rarely lethal for vertebrates without other immuno-compromising conditions [2]. However the recent discovery of deadly fungal skin infections in natural vertebrate populations (e.g., [3]) raises a number of questions about how a pathogen localized to the skin can lead so quickly to death and threaten entire populations.

One of the most dramatic examples of the impact of pathogenic fungi on vertebrate populations is the chytrid *Batrachochytrium dendrobatidis* (hereafter *Bd*), which has been implicated in global amphibian declines. This pathogen, discovered and described only 10 years ago [4], infects hundreds of amphibian species worldwide [5]. *Batrachochytrium dendrobatidis* is an aquatic fungus and attacks the keratinized epithelial cells of adult amphibian skin [6]. Chytridiomycosis, the disease caused by *Bd* is often fatal, and is the clear cause of catastrophic reductions in many frog populations around the world (e.g., [7], [8]).

Although some clinical, physiological, and immunological responses of frogs suffering from chytridiomycosis have been described [6], [9], [10], remarkably little is known about the mechanisms involved in amphibian response to *Bd*. At the clinical level, frogs suffering from chytridiomycosis exhibit hyperkeratosis with epidermal hyperplasia and excessive skin shedding [6], [11]. Recent work has demonstrated that frogs with severe *Bd* infections exhibit decreases in electrolyte concentrations, plasma osmolality and blood pH [9]. Osmotic and electrolyte imbalances help explain the proximate cause of frog mortality due to chytridiomycosis, but much remains to be discovered about the physiological effects of *Bd* infection. Similarly, the immune response to *Bd* is not well understood. A number of studies have shown that the anti-microbial peptides (AMPs) secreted by amphibians can inhibit *Bd* growth *in vitro* and may play a role in resistance to infection (e.g. [10]). Although AMPs, as an important component of the innate immune system, represent a potential first line of defense against *Bd*, their direct role in combating *Bd* infection in nature remains to be demonstrated. More broadly, there is no published evidence for an adaptive immune response in frogs exposed to *Bd*.

Many other fungal pathogens elicit a robust adaptive immune response in their vertebrate hosts (see review in [12], [13]), and there is no reason to suspect *a priori* a diminished role for adaptive immunity to *Bd*. For example, the cell types involved in mammalian response to fungal skin pathogens (dermatophytes) are similarly found in the skin of *Xenopus laevis* [14]–[16] and would be expected to mount a similar localized inflammatory response. Several recent reviews have called for further investigation of the role of adaptive immunity in frog response to *Bd* [17], [18]. Moreover, given the considerable cross-talk between elements of innate and acquired immunity, it is productive to take a comprehensive approach to studying frog immune response to *Bd*. Such a comprehensive characterization of host response to pathogen exposure can be achieved with genome-wide expression studies [19], [20].

Here we present a global approach to understanding frog responses to chytrid exposure by using whole genome transcription assays in *Silurana tropicalis* (formerly *Xenopus tropicalis*, [21]). *Xenopus* and *Silurana* frogs have been used for many decades in immunological investigation (e.g., reviewed by [14], [22]). Additionally, the complete genome of *S. tropicalis* has now been sequenced, enabling genome-scale approaches to studying the response to pathogen exposure. We document gene expression changes in multiple tissues at multiple time-points for frogs experimentally exposed to *Bd* under controlled laboratory conditions. We present data on the genetic pathways perturbed in response to *Bd* and discuss the implications for understanding disease-related amphibian declines.

## Materials and Methods

### Animal husbandry

Adult *Silurana tropicalis* were used for infection experiments, and frogs were housed individually for the duration of the experiment. Twice per week, frogs were fed *ad libitum*, and tank water was changed. Stock colonies of S. tropicalis were maintained at 25°C. However, experimental inoculations and observations were executed at 18°C. It is important to note that this temperature regime may be suboptimal for amphibian immune response; however, there are several reasons that this first study was conducted at this temperature. First, we were interested in the physiological changes associated with fatal *Bd* infections, and we found a stronger reaction to *Bd* exposure at cooler temperatures during preliminary trials. Second, many catastrophic *Bd*-related declines in natural populations occur during cooler months and in cooler montane habitats [23], [24], therefore understanding the response to *Bd* exposure at non-preferred temperatures is critical. Third, we prioritized conducting experiments within the optimal growth temperature for *Bd* [25] to support ongoing work connecting gene expression patterns of both host and pathogen.

Experimental frogs were acclimated to progressively cooler temperatures over the course of one week and then allowed to acclimatize for an additional week at 18°C. A swabbing method was used for RT-PCR tests to ensure that all experimental animals were chytrid-free prior to inoculations [26]. Inoculum was prepared by growing *Bd* on 1% agar, 1% tryptone plates for 2 weeks. Chytrid plates were then flooded with water, and frogs in the exposed group were held overnight in a 100 mL water-bath inoculation. Exposures were repeated with each tank water change. Frogs in the control group were treated identically but received only sham inoculations (no *Bd* exposure). Each control frog was sacrificed at the same time as an infected frog from the same clutch, of the same sex, and of similar size and weight. Resulting paired control-infected samples were handled simultaneously throughout the experiment to ensure comparability. Frogs were swabbed prior to euthanasia to quantify *Bd* load.

Frogs were euthanized by decapitation at two time-points after initial *Bd* exposure. The “early” group was euthanized 72 hours after first exposure to *Bd*. The “late” group was euthanized once clinical symptoms of chytridiomycosis were observed between 10 and 21 days following initial exposure. We chose to sample the “late” group based on near-death symptoms rather than at a fixed time-point because we were interested in the genetic perturbations associated with the final stages of disease progression. Many of these late group frogs exhibited excessive skin shedding and extreme lethargy. The late group frogs exhibited higher infection loads than early group frogs (see Results), so the different time points correspond with infection intensity. Dorsal skin, liver, and spleen tissues were flash frozen and stored at −80°C.

### Microarray Design

We used a custom NimbleGen microarray for *S. tropicalis* based on full length coding sequence (CDS) and expressed sequence tags (ESTs) from the Joint Genome Institute (JGI; available at http://genome.jgi-psf.org/Xentr4) and Gurdon Institute (available at http://informatics.gurdon.cam.ac.uk/online/xt-fl-db.html). The microarray contained 380,856 60-mer probes representing a possible 113,986 expressed transcripts (probe sets). Fifty-six percent of probes were designed from CDS and 44% were designed from ESTs. Probe sets were defined as all probes that mapped to a single transcript, which in turn mapped to a single gene. Each probe set consisted of 1 (44% of probe sets), 3 (31% of probe sets), or 8 (25% of probe sets) probes. Although probe sets consisting of only one probe were included on the array, none met our statistical criteria for differential expression (see Results). Both JGI and Ensembl (http://www.ensembl.org/Xenopus_tropicalis) annotation of the *S. tropicalis* genome were used to assign functional categories to genes.

### Experimental design

For each microarray, a competitive hybridization was performed, in which two samples were labeled with different Cy dyes and hybridized to the same chip to provide a direct comparison between control and infected frogs. A total of 12 chips were analyzed from 24 samples comprising 2 conditions (control and infected), 2 tissues (dorsal skin and liver) and 2 time-points (early and late). Three biological replicates (i.e., individual frogs) were used for each condition and tissue at each time-point. Cy3 and Cy5 dyes were alternately used for labeling each tissue at each time-point (i.e., dye balanced) in order to account for potential dye bias.

In addition to the 12 liver and skin chips, a single chip was run to gain a preliminary understanding of gene expression patterns in the spleen. Because *S. tropicalis* spleens are small and a large amount of RNA is needed for whole genome assays, our ability to assay whole genome patterns of expression in the spleen was limited. We were able to compare expression patterns for control versus infected frogs early in infection by pooling spleens from frogs sampled at the early time-point. However frogs late in infection were often so dehydrated that we could not collect their spleens, and thus we did not analyze gene expression in the spleen at the late time-point.

### Molecular Methods

RNA was extracted with a Qiagen RNeasy Mini Kit (Qiagen,Valencia, CA) with the standard protocol and a DNase digestion. Double-stranded cDNAs were synthesized using Invitrogen's SuperScript cDNA Synthesis Kit (Invitrogen, Carlsbad, CA). cDNA samples were fluorescently labeled with custom Cy3 and Cy5 labeled oligonucleotides from TRILink BioTechnologies (TRILink BioTechnologies, San Diego, CA). A 16-hour hybridization was conducted at 42 degrees in a Hybex hybridization chamber (SciGene, Sunnyvale CA). Chips were washed and then scanned on a GenePix Professional 4200 Scanner (MDS Analytical Technologies, Sunnyvale, CA). We corroborated chip-based gene expression patterns for a subset of genes with RT- PCR using SYBR Green (Qiagen, Valencia, CA) and an ABI 7300 (Applied Biosystems, Foster City, CA). We quantified gene expression of target genes of interest relative to reference genes that exhibited constitutively high expression. Reference genes used were beta actin (295226) and proteasome activator 28 (294819) (throughout parenthetical gene numbers correspond to JGI gene IDs). Results for four genes of interest [cytochrome P450 (448177), keratin (178963), proteinase inhibitor (345734), and apoptosis regulator (416168)] are shown here (see Results). Mean and standard deviation of at least three control and three exposed individuals with technical replicates are presented. Results were the same using both reference genes; in all cases fold changes between naïve and exposed frogs estimated using RT-PCR were in the same direction and at least the same magnitude (often greater) than the chip-based estimates.

### Data Analysis

NimbleScan software (NimbleGen. Madison, WI) was used to align a chip-specific grid to control features and extract raw intensity data for each dye and probe. Raw intensity data was read into the R computing environment using the LIMMA package for two-color array analysis and checked for quality. Chip pseudo-images were created for each array and dye and visually verified not to contain any spatial artifacts. Further, chip intensity distributions and MA-plots were compared and checked for any unusual global patterns. Each array was then normalized using loess normalization and then across arrays using the A-quantile normalization procedure [27]. Each probe set was then summarized using the median polish procedure as described in Irizarry et al. [28]. The median polish procedure is a robust method for summarizing all probes contained within each probe set to a single expression value for each gene taking into account individual probe effects. Probe sets with low levels of expression variation across all samples (IQR<0.5) were removed from further analysis, leaving 19,719 probe sets remaining in the study. Differential expression was then assessed using a linear model with an empirical Bayesian adjustment to the variances [29], and comparisons of interest were extracted using contrasts. The Benjamini and Hochberg (BH) method was used to control for the expected false discovery rate given multiple tests [30]. Probe sets were considered differentially expressed with a BH adjusted p-value of <0.05 and a raw-fold change greater than 1.5. Because only one chip was run for the pooled spleen samples, only preliminary analyses were performed for the spleen. The single spleen array was preprocessed in the same manner as the dorsal skin and liver arrays. Relative gene expression differences were then calculated for each probe set as the log difference in signal between infected spleens and control spleens. Genes with at least 2-fold differences in expression were considered the most likely candidates for further study. All microarray data is publicly available in accordance with MIAME (GEO accession numbers GPL8767, GSE16852), and the complete list of differentially expressed probe sets is provided in Table S1.

## Results

**Figure 1:**
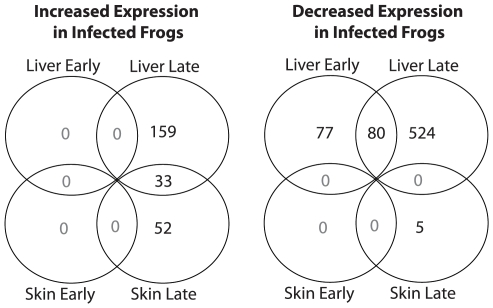
Summary of significant changes in gene expression between *Bd*-exposed and naïve control frogs. Numbers of genes with increased and decreased expression in infected frogs are shown for the skin (the site of infection) and the liver (an immunologically and physiologically important organ). Data are summarized for two time-points: early (3 days after initial exposure to *Bd*) and late (when clinical symptoms of chytridiomycosis are evident).

**Figure 2:**
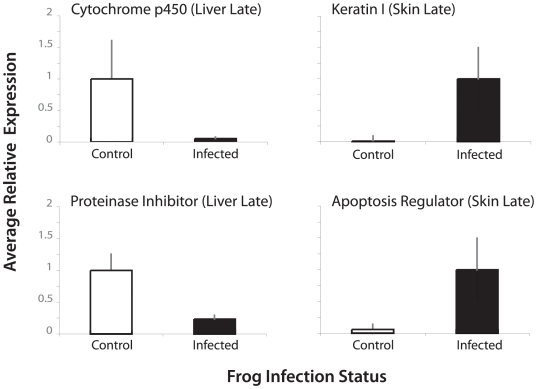
Validation of chip-based gene expression patterns with RT-PCR. Average expression is shown for several genes of interest relative to an endogenous control (beta actin). Mean and standard deviation are shown for biological replicates. In all cases RT-PCR results were in the same direction as array-based results and of comparable (or greater) magnitude.

There were 923 probe sets corresponding to unique *S. tropicalis* transcripts with statistically significant patterns of differential expression in at least one tissue for at least one time-point (Fig. 1). Of these probe sets 42% contained 3 probes and 58% contained 8 probes. RT-PCR confirmations of gene expression patterns for a subset of genes of interest are shown in Figure 2. At the early time-point (72 hours after the first *Bd* exposure) the transcriptional response to *Bd* exposure was surprisingly weak. At this early time-point a per-swab average of 3,500 *Bd* zoospore equivalents was estimated by RT-PCR. There were 157 genes with decreased expression in exposed frogs (relative to naïve frogs) in the liver 3 days after initial exposure, but no genes showed significant increases in expression in the liver or skin early in infection. In the spleen early in infection, 120 genes showed significant differences in expression, with 99 genes showing increased expression and 21 genes showing decreased expression. At the late sampling point (when clinical symptoms of chytridiomycosis were manifest) there was a stronger global transcriptional response in exposed frogs. At the late sampling point a per-swab average of 51,500 *Bd* zoospore equivalents was estimated by RT-PCR. In the liver, 604 genes had significant decreases in expression, while only 192 showed increases. In the skin, 85 genes showed increases, while only 5 genes showed decreased expression.

### Immune response

To gain an overall picture of the expression changes observed in immune-related genes, the probe set annotations were queried with the keyword “immune”. Significant changes in gene expression were observed in only 3 out of 62 probe sets containing immune function Gene Ontology (GO) annotations. To identify additional genes related to immune function that did not contain GO immune annotation, the dataset of differentially expressed genes was manually interrogated to categorize genes related to immunological responses.

The expression changes in immune related genes mirrored the overall patterns of expression differences across the genome, with decreased expression of immune-related genes in both the skin and liver of *Bd*-exposed frogs (Table 1). Notably, a number of genes in the complement pathway showed decreased expression in exposed frogs. At the early time-point, 10 complement pathway genes (of 38 total) showed decreased expression in exposed frogs including C1r, C4, C9, factor H, factor I, and alternative pathway C3/C5 convertase (Table 1). At the late time-point, 5 complement pathway genes showed decreased expression including C1r, C9, and classical pathway C3/C5 convertase (Table 1). No complement-related genes showed increased expression in exposed frogs.

**Table 1:** Summary of immunological and physiological genes of interest with significant differences in expression between control and infected frogs.

Function	Tissue	Time-Point	Average Fold Change	JGI Gene ID	Ensembl Gene ID
Coagulation factor	liver	early	−4	279909, 293803, 330213, 341521, 341530, 341561	ENSXETG00000002964, ENSXETG00000002769
Coagulation factor	liver	late	−5	147254, 279909, 292258, 292259, 293803, 330213, 341521, 341530, 341561	ENSXETG00000002964, ENSXETG00000002769, ENSXETG00000003472, ENSXETG00000025492, ENSXETG00000025493
Complement component (alternative pathway)	liver	early	−6	308546, 327877, 420558	ENSXETG00000005261, ENSXETG00000010935, ENSXETG00000024110
Complement component (membrane attack complex)	liver	early	−6	282192, 295244	ENSXETG00000003532, ENSXETG00000006766
Complement component (classical pathway)	liver	early	−3	399987, 471107	ENSXETG00000005203, ENSXETG00000027636
Complement component (classical pathway)	liver	late	−2	399976, 399987,400010, 420551	ENSXETG00000010930, ENSXETG00000023914, ENSXETG00000027636
Cytochrome Cyp450	liver	early	−3	113474, 297805, 325065, 448177, 464259	ENSXETG00000005868, ENSXETG00000006924, ENSXETG00000013846, ENSXETG00000016349, ENSXETG00000024804, ENSXETG00000027605
Cytochrome Cyp450	liver	late	−4	>50 genes, see Table S1	>50 genes, see Table S1
Epoxide hydrolase	liver	late	−4	126998, 152658, 398826	ENSXETG00000017581, ENSXETG00000022654, ENSXETG00000028064
Heat shock protein 70	liver	late	4	268191, 287936, 361977, 456096	ENSXETG00000006714, ENSXETG00000008828, ENSXETG00000010075, ENSXETG00000016838
Heat shock protein 70	skin	late	6	361977, 456096	ENSXETG00000006714, ENSXETG00000008828
Keratin, type I	skin	late	10	178963	ENSXETG00000012482
NF-kappa inhibitor	liver	late	3	309572, 396637, 309732	ENSXETG00000006998, ENSXETG00000012114
Ornithine decarboxylase	skin	late	18	196068, 284956, 295202	ENSXETG00000003519, ENSXETG00000007373
Small chemokine, interleukin 8	liver	late	−3	295905	NA

Because multiple genes with similar function were often perturbed, fold change is given as an average for all differentially expressed genes in each category. Genes with increased and decreased expression in *Bd*-exposed frogs show positive and negative fold changes, respectively. See text for additional genes of interest and Table S1 for a complete gene list of differentially expressed genes.

A small number of immune-related genes were up-regulated in *Bd*-exposed frogs, but the up-regulation of most of these genes would have a net-inhibitory effect on immune response. Specifically, the function of immune genes with increased expression were generally modulatory (or regulatory) in signal transduction pathways including suppressor of cytokine signaling 3, NF-kappa-B inhibitor zeta, and A20-binding inhibitor of NF-kappa-B activation 2 (Table 1). Inhibition of the transcription factor NF-kappa-B is noteworthy given its important role in regulating many genes involved in the production of an immune response.

Several genes with increased expression in *Bd*-exposed frogs provide evidence for some activation of innate immune responses against *Bd* (Table 1). We observed increased expression in phagocyte-associated genes including neutrophil cytosolic factor 4, neutrophil collagenase precursor, cytochrome b-245 light chain, arginase I, and lysozyme (Table 1). However, nearly all of these increased expressions occurred at the late time-point rather than early in infection when activation of innate defenses would be expected. In addition, some evidence for innate and adaptive immune response at the early time-point was obtained from our preliminary spleen data. We observed increased expression in the spleens of *Bd*-exposed frogs for a small number of chemokine (295161, 457013) and neutrophil (449373) genes. Some additional immune function genes had weak evidence of activation in the spleen early in infection. Although fold-changes were lower than our cutoff, all probes in the following probe sets showed increased expression in the spleen: interleukin-17A/F-like (146326) and its precursors (e.g., 144803), MHC complex proteins (e.g., 443195), myd88 (a key molecule in relaying the signal from toll-like receptors, 294414), and complement (e.g., 296194, 194000). It is important to note that the signature of increased expression in sick frogs was not consistent across all genes in these categories. Furthermore, a large number of immune function genes that we expected to be differentially expressed during an immune response to *Bd*, did not show any significant changes in expression in *Bd*-exposed frogs. Table 2 illustrates examples of genes and gene classes that we expected to be differentially expressed but were not.

**Table 2:** Examples of genes expected to be involved in a robust immune response that showed unchanged expression between infected and control frogs.

Description	JGI Gene_ID	Ensembl.Gene.ID
Anti-microbial peptide (AMP)	287907	NA
Anti-microbial peptide (AMP2)	109728	ENSXETG00000022894
Cathelicidin (AMP)	193370	NA
Heat shock protein 90 family (HSP90)	e.g., 124362, 201402, 293016, 295840, 300776, 350432, 378380, 468257	ENSXETG00000005359, ENSXETG00000010251, ENSXETG00000010896, ENSXETG00000019484, ENSXETG00000001738
Indoleamine 2,3-dioxygenase	312615	ENSXETG00000025783
Interferon gamma (IFNgamma)	177216	NA
Interleukin 1 (IL-1)	119533	NA
Interleukin 17 (IL-17)	300117	ENSXETG00000020860
Interleukin 8 (IL-8)	295161, 295905, 460999	NA
Major histocompatibility complex (MHC) class II alpha and beta	443195, 151191, 371515, 446178, 152977	ENSXETG00000025586, ENSXETG00000025590, ENSXETG00000017066
Major histocompatibility complex (MHC) class I	386203, 295817, 386304, 386189, 386261	ENSXETG00000014445, ENSXETG00000017065, ENSXETG00000009077
T cell and B cell marker (cd45)	308517	ENSXETG00000000316
T cell marker (cd28)	149891	NA
T cell marker [Thy1 (CD90)]	452827	NA
Toll-like receptor (TLR5)	459490	ENSXETG00000015027
Tumor necrosis factor alpha (TNFalpha)	379518	NA

### Physiological Response

One of the most dramatic and consistent signatures observed in our experiment was the decreased expression of a large number of cytochrome p450 (P450) superfamily genes in exposed frogs (Table 1). Fifty-one unique P450 genes (out of 70) showed decreased expression in the liver of infected frogs late in infection. Six of these genes already showed decreased expression in the liver early in infection, although the average fold-change between sick and healthy frogs was more dramatic later in infection. Aside from changes in P450 genes, we observed decreased expression of several epoxide hydrolase genes (126998, 152658, 398826) late in infection in the liver, which are similarly involved in cellular detoxification. One of these genes (398826) also showed decreased expression in the liver at the early time-point. Another class of genes with a net decrease in expression is related to blood coagulation. We observed decreased expression at both the early and late time-point in the liver of coagulation factors, thrombin, and plasmin genes (Table 1). Other than fibrinogen-related genes (which had a mixed pattern), coagulation-related genes with higher expression in infected frogs were generally inhibitory (e.g. 287154) so the net effect of deactivation of coagulation genes appears consistent. Additionally, one important marker of hepatic anoxia; HIF-1 (hypoxia induced factor, 151770) showed increased expression in the late liver samples of infected frogs, suggesting a reduction in oxygen delivery and permeation.

We were also interested in determining whether *Bd*-exposed frogs exhibited signs of acute and/or chronic stress. All of the heat shock proteins we observed with significant changes in expression (9 out of 69 annotated genes) showed higher levels of expression in infected frogs. Increased expression of HSP70, HSP20 and HSP DnaJ were evident at the late time-point in both the liver and skin (Table 1). In the spleen early in infection we also observed increased expression of genes with HSP70 (174350, 287936, 445165) and HSP90 (468257) domains. Other than HSP72 and HSP90, we did not observe the up-regulation of many other HSPs typically associated with acute stress response (i.e., HSP9, HSP23, HSP26, HSP27) in any tissue at any time-point.

We also observed perturbations in genes involved in cellular integrity in the skin. There was more than a 10-fold increase in expression in the keratin I gene (178963) in frog skin late in infection. Additionally, late in infection we observed higher expression in *Bd*-exposed frogs of genes coding for matrix proteins (165447) growth factors (163909), connexin (175537), collegen and fibrinogen (335826, 317405, 336019, 166058), and ornithine decarboxylase genes (284956, 295202, 196068). There were no genes in these categories with decreased expression in the skin, nor were there any changes in these genes early in infection. Somewhat surprising was the fact that no members of the caspase family were significantly altered in the infected frog skin; this would seem to indicate that neither the intrinsic nor the extrinsic apoptotic pathways were activated in response to cutaneous colonization by *Bd*.

## Discussion

We have evaluated whole genome expression profiles of *S. tropicalis* exposed to the pathogenic chytrid fungus, *Bd*. We characterized changes in gene expression globally for predicted transcripts throughout the genome at two time-points after *Bd* exposure (early and late) at the site of infection (skin) and important immune organs (liver and spleen). We observed a very weak transcriptional response 3 days after exposure (Fig. 1). Notably, there was not a single gene in the liver or skin with a significant increase in expression at the 3-day time-point suggesting that frogs were not recognizing and mounting an appropriate early response to infection in our experiment.. The transcriptional response late in infection was more dramatic (Fig. 1). But again the vast majority of perturbed genes showed decreases in expression in sick frogs compared to uninfected control frogs kept at the same temperature. This extensive negative effect on gene expression only in infected animals is likely linked to infection rather than just an effect of suboptimal temperature on host immune defenses. RT-PCR validations confirm the array results, and the consistent expression of transcriptional regulators indicates that we are not merely observing global transcriptional suppression. Below we discuss the implication of our findings with particular emphasis on the a) strength and nature of the immune response and b) physiological changes during disease progression.

### Immune Response

A typical robust immune response to a skin infection would include both innate and adaptive components of the immune system at the site of infection as well as in main immune organs such as the liver and the spleen. The initial innate response would be expected to include activation of macrophages, neutrophils, and Langerhans cells as well as pro-inflammatory signaling to recruit more effector cells (leukocytes). The local immunological signals in the skin would be expected to involve activation of cell surface receptors (e.g., toll-like receptors and MHC molecules), inflammatory cytokines, chemokines, cytokine-induced proteins, and anti-microbial peptides. During the early phase of immune activation we would expect to observe increased hepatic gene expression of acute phase gene-encoded proteins including C-reactive protein, complement pathway genes, and coagulation genes. As the infection progresses, the adaptive component of the immune response would be expected to be activated. The signature of an adaptive response would entail cellular markers of lymphocyte maturation/activation (e.g., B and T cell receptors and co-receptors, activation-induced cytidine deaminase, antibodies), antigen presentation (e.g., LMP7, TAP1, cathepsines, MHC molecules) as well as cytokine production (e.g., IL-2, IL-4).

In mammals, the immune response to dermatophytes (pathogenic fungi that cause ailments such as ringworm) may present an analogous scenario from which to derive expectations for amphibian response to *Bd*. The response to dermatophytes is characterized by a delayed-type hypersensitivity response (reviewed in [13]). To initiate the process, Langerhans cells gather antigens and move to peripheral lymphoid tissue (e.g. spleen) and present the antigens to T lymphocytes. At the site of infection, T lymphocytes mediate the recruitment of phagocytes via cytokine-driven induction of a localized inflammatory response. Studies on *Xenopus laevis* have shown the presence of these cell types in the skin, so it is reasonable to expect a comparable response [14], [15].

Our results show that very few components of a typical innate or adaptive immune response occurred in *S. tropicalis* exposed to *Bd*, in fact most immune related genes showed decreased expression in infected frogs. Nearly all of the genes that we hypothesized to play a role in controlling *Bd* infection showed no expression change between exposed and naïve frogs (Table 2). For example in the liver and skin, there was no activation of toll-like receptors (which could play a role in pathogen recognition), no change in known antimicrobial peptide genes (although not all AMPs were represented on our chip because some sequences were shorter than our 60-mer probes), and no change in MHC class I or II genes, or genes involved in antigen presentation (e.g., LMP7, TAP1 and 2, cathepsines). Only phagocyte-associated genes exhibited increased expression in infected frogs, and only at the late time-point (Table 1). It is dramatic that of dozens of classes of immune function genes that are usually activated in the liver, phagocyte-associated genes are the only class showing a positive response. The activation of phagocytosis itself is not evidence of a robust immune response given that these genes could be activated in response to other cellular changes in the liver, especially at late stages of infection. There were a handful of immune-relevant genes with increased expression in the spleen. For example, several MHC and interleukin-related genes showed higher expression in *Bd*-exposed frogs. Although the trends in the spleen data may suggest some activated immune responses, there were only a handful of immune-function genes with consistent signature of increased expression, and robust statistical statements are precluded by the lack of replication for the spleen chip. It is therefore critical for future studies to further dissect patterns of immunogenetic response in replicated spleen samples.

In contrast to the few immune genes showing increased expression, a large number of immune genes showed decreased expression in exposed frogs. The strongest negative signal for immune-related genes was observed in the complement pathway. At both early and late sampling time-points we observed reduced expression of complement pathway genes in the liver. The consistency of the observation may suggest that complement suppression is a characteristic feature of chytridiomycosis in *S. tropicalis*. The cascade of events leading to complement suppression remains to be determined but may result from several causes. First, if one complement pathway is up-regulated, a compensatory down-regulation may be expected in an alternate pathway. However, we see decreased expression in complement genes in both the classical and alternate pathways suggesting this explanation is unlikely. Second, it is possible that the down-regulation of complement represents exhaustion from an earlier up-regulation, but again this seems unlikely given that we observed decreased expression of complement-related genes as early as 72 hours after the initial exposure. Finally, decreased expression of complement could be a direct result of an inhibitory signal from *Bd*. As of yet we have no direct evidence for active immune suppression by *Bd*, but other fungal pathogens have been associated with complement evasion. For example, human pathogenic fungi *Aspergillus fumigatus* and *Candida albicans* evade immune attack by binding complement regulators in order to suppress complement effector molecules [31]. Regardless of the mechanism by which complement and other elements of the immune system are suppressed, it is clear that the net effect will be detrimental for amphibian immunity given the importance of complement in B cell and antibody responses. The decreased expression of complement could also affect complement-enhanced phagocytosis, particularly given the expression changes observed in phagocyte-associated genes. Overall, we expect the observed reduction in complement activity to be problematic, not only for mounting an appropriate response to an existing assault from *Bd*, but also for responding to future pathogenic insults.

There are three non-mutually exclusive hypotheses for the lack of robust immune response observed in our experiments: 1) immune evasion/suppression by *Bd*, 2) reduced immune response in highly susceptible frog species, and 3) reduced immune response under certain environmental conditions. First, the lack of a robust immune response may indicate immune evasion/suppression strategies by *Bd*. For example, *Bd* may be able to sequester itself from immune receptors during infection in order to avoid immune attack. During intracellular stages of infection, *Bd* may be able to avoid immune recognition in the protected environment of the skin layer (keratinocytes). For example, several other intracellular pathogens are known to employ this strategy including *Histoplasma capsulatum*, which is a dimorphic fungal pathogen that proliferates inside mammalian macrophages [32]. In another example, fungal pathogen *Coccidioides posadasii* secretes a metalloproteinase that mediates immune evasion by digesting the pathogen's own surface antigens in order to avoid recognition [33]. Another possibility is that *Bd* suppresses the immune response by interfering with immune signaling. Other fungal pathogens are known to display or secrete molecules that disable host defense pathways. For example, dimorphic fungal pathogen *Blastomyces dermatitidis* produces an adhesion molecule (BAD1) that binds to a host complement receptor in order to suppress production of pro-inflammatory cytokine TNF-α [34]. Prior studies of *Bd* gene expression throughout development identified the differential expression of several genes with sequence similarity to genes thought to be involved in pathogen evasion of vertebrate immune systems [35], but further research is necessary to evaluate which *Bd* secreted proteins may interact with the host immune system.

Second, the reduced immune response may be characteristic of highly susceptible frog species. There is a wide variation of susceptibility of frogs to chytridiomycosis and different outcomes to *Bd* exposure have been observed in both laboratory and natural settings. In lab infection experiments mortality can range from zero to 100% depending on the species [36], [37]. Similarly, in nature, some species experience dramatic *Bd* related declines while others persist with moderate *Bd* loads while apparently suffering no ill effect. *Silurana tropicalis* is a susceptible species, so our observations are likely not to be generalized across amphibian species. Differences in species susceptibility to *Bd* are hypothesized to be related to differences among species in the production of antimicrobial peptides [10], [38], the occurrence of bacterial commensals with anti-fungal properties [39], and behavioral characteristics [40]. However, there is scant data exploring differences in innate or adaptive immune response among species with different susceptibilities, and it is possible that some susceptible species do not mount effective innate and/or adaptive immune responses, while other more resistant species do. Immunological studies on closely related resistant/susceptible species pairs (e.g., *X. laevis* and *S. tropicalis*) are a priority for continued research.

Third, the damped immunogenetic response observed for *S. tropicalis* in this study is unlikely to be generalized across all environmental conditions. Importantly amphibian immune responses can be strongly affected by temperature (e.g., [41]). Further, laboratory infection experiments (not in *Silurana*) suggest that differences in temperature on the order of 5–10 degrees C can affect mortality rate [24]. Although our study was not conducted at the low temperatures known to have dramatic impacts on frog immune function (e.g., 5 degree C [41]), the temperature regime we used was outside the preferred temperature profile of *S. tropicalis*. In *X. laevis* 18 degree C is cold enough to have inhibitory effects on T cell proliferation [42] and IgM-IgY switching [43]. Although it may be argued that the temperature at which the frogs were infected here was not optimal for a potent immune response, the fact we observed many immune genes down-regulated and not just unchanged compared to control animals (at the same temperature) is indicative of a more complex phenomenon linked to the infection. Additionally, because *Bd* related die-offs often occur in colder environments and colder seasons [23], [24], it is biologically relevant to understand frog response to *Bd* when not operating under optimal immunity. Follow-up studies should consider immunogenetic response of *S. tropicalis* under different temperature regimes and endeavor to further understanding the interaction between chytridiomycosis and environmental conditions that may decrease immunocompetence.

### Physiological Response

While no specific physiological effect, outside of immunological response, was predicted from *Bd* exposure, we expected a pronounced transcriptional response in the hepatic and cutaneous tissues considering the lethality of *Bd* colonization. The overall patterns observed for physiologically relevant genes were complex but point to several important physiological changes in *Bd* infected frogs. First, we observed a strong signature of chronic stress in *Bd*-exposed frogs. Genes coding for heat shock proteins were up-regulated early in infection in the spleen (HSP70, HSP90) and late in infection in both the liver and skin (HSP70, HSP20, HSP90 and HSP DnaJ). The chronic stress response observed late in infection could be due to any number of physiological changes in frogs close to death, and does not necessarily reflect a signature particular to chytridiomycosis. However, the consistent increase in expression for HSPs just three days after *Bd* exposure does suggest a direct stress response to *Bd*. It is interesting to note that we observed the perturbation of only some of the stress-related HSP genes and that some of the classical markers of acute stress response were unaffected. However some inducible markers like HSP72, which is often increased during microbial infection, and HSP90 were perturbed. We are confident that the increase in stress-related genes was above and beyond any stress due to experimental conditions (e.g., captivity, handling, temperature) as the stress related genes showed on average four-fold greater expression in *Bd*-exposed versus control frogs.

We also observed genetic changes that can help explain some of the clinical symptoms of chytridiomycosis in the skin. The large increase in expression in the type I keratin gene in skin late in infection is particularly interesting. This gene is an ortholog of human keratin 1, which is known to be perturbed in humans with the ailment epidermolytic hyperkeratosis [44]. Although this disease and chytridiomycosis have different etiologies, it is intriguing that similar phenotypes result (e.g., expansion and irregularity of the stratum corneum). There is additional molecular evidence that the skin is damaged and trying to repair with increased expression of genes involved in cellular remodeling of the skin and tissue integrity (e.g., matrix proteins, connexins, collagens, fibrinogens). The up-regulation of genes involved in cell growth and proliferation (e.g., growth factors and ornithine decarboxylase), may contribute to the observed expansion of the stratum corneum and the sloughing response of frogs with chytridiomycosis. Whether this skin shedding is a physiological strategy of frogs to shed *Bd* infection or a response to *Bd* toxins requires further study. We did not observe perturbations of genes that could be involved in cytoxic or antigenic response in the skin, and thus our skin data do not directly indicate the production of toxic compounds by *Bd*. Further, the absence of significant changes in the skin in the expression of caspase genes, typically altered in response to cell damage and important mediators of the apoptotic cascade, was particularly surprising given the colonization by *Bd*.

However, changes in another class of genes important in cellular detoxification may point to the possibility of *Bd* toxicity. We observed the down-regulation of a large number (N>50) of cytochrome P450 genes (and relatedly epoxide hydrolase genes) in the liver. P450 genes are known to be important in detoxification [45], and their down-regulation may be indicative of exhaustion (although we did not observe an early up-regulation of these genes). Alternatively, their down-regulation may be indicative of *Bd*-derived toxins, as perturbation of liver P450 function can be endotoxin-mediated in other systems [46]. Perhaps more importantly than the cause of decreased expression is the effect of decreased expression of this gene family, which is expected to have physiological consequences in pathways related to cellular metabolism and detoxification. Systemic mycoses have been shown to alter the expression of P450 genes in mammalian livers, in some instances facilitating host defense while in others associated with the etiology of the disease [47]. Given the diversity of metabolic pathways that P450 gene products are involved with, we can suggest a broad-spectrum alteration in the hepatic capacity for oxidative metabolism associated with the down-regulation of these genes. The consequences of changes in Cyp450 function, particularly for amphibians, are likely to be severe because they are in close contact with environmental contaminants, and perturbations in this gene family may affect the ability of frogs to fight off future insults.

Finally, we observed a number of changes in blood coagulation factors in the hepatic transcriptome. It is possible that these are related to the subjective assessment that the *Bd* infected frogs were dehydrated, but we did not have any direct measures of hydration such as packed cell volume, osmolality or hematocrit to directly confirm this observation. Although the cause of alteration in expression of these hemostatic markers is uncertain, it is interesting to note that similar changes are observed in scenarios of known diminished liver function in mammals, such as cirrhosis [48] and post-transplantation [49]. Thus there is some indication that the livers of infected frogs were not fully functioning, however we did not observe perturbation of any classical markers of liver failure. Another important change in the livers of infected frogs was the up-regulation of hypoxia-induced factor-1. This gene is a potent and acute marker of low oxygen delivery and may indicate alteration in the efficacy of cutaneous blood gas exchange in the *Bd* infected late stage frogs. Prior studies have not reported any significant changes in measures of pCO_2_ in *Bd* infected frogs [9], but induction of hypoxia-induced factor-1 may indicate changes in skin gas exchange associated with *Bd* colonization. Of course transcriptome data should not be used as the sole index of response to infection, and additional work is necessary to link gene expression data to organismal level observations. But hypoxia-induced factor-1 is a sensitive marker for alterations in oxygen utilization or delivery and has broad-spectrum downstream targets [i.e., implicated in activating genes involved in angiogenesis, apoptosis, mitochondrial biogenesis, anaerobic metabolism (e.g. [50])]. Therefore patterns observed at this gene are worth further study.

Although our data do not determine definitively the proximate cause of *Bd* morbidity, we provide insight into which genetic systems are perturbed during infection. First, we show that the activation of the immune system is weaker than expected. Second, we provide information about the molecular mechanisms underlying stress response and skin sloughing. Finally we provide some insight into *Bd* pathogenesis with data suggesting that *Bd* may compromise immune response/detection. These baseline observations make possible future studies to understand response of frogs to *Bd* in natural settings and to compare populations and species with different levels of resistance to *Bd*.

## Supporting Information

Table S1 Complete list of genes with significant differences in expression between naïve and *Bd*-exposed frogs. Table columns correspond to gene identifiers (NimbleGen, Joint Genome Institute, and Ensembl), number of probes in probe set, summary of tissues and time points for differential expression, log2 fold-changes for tissues and time points with significant differences in expression, and functional annotation (e.g., InterPro, Gene Ontology, euKaryotic Orthologous Groups).
(0.40 MB XLS)
